# Histone Deacetylase (HDAC)-1, -2, -4, and -6 in Uveal Melanomas: Associations with Clinicopathological Parameters and Patients’ Survival

**DOI:** 10.3390/cancers13194763

**Published:** 2021-09-23

**Authors:** Georgia Levidou, Pawel Gajdzis, Nathalie Cassoux, Piotr Donizy, Christos Masaoutis, Malgorzata Gajdzis, Sophie Gardrat, Alexandros Pergaris, Eougken Danas, Jerzy Klijanienko, Stamatios Theocharis

**Affiliations:** 1 First Department of Pathology, National and Kapodistrian University of Athens, 15772 Athens, Greece; georgia.levidou@klinikum-nuernberg.de (G.L.); cmasaout@med.uoa.gr (C.M.); alexperg@yahoo.com (A.P.); danaseugene@gmail.com (E.D.); 2 Department of Pathology, Paracelsus Medical University, 90419 Nuremberg, Germany; 3 Department of Pathology, Curie Institute, 75005 Paris, France; pawel.gajdzis@umed.wroc.pl (P.G.); jerzy.klijanienko@curie.fr (J.K.); 4 Department of Clinical and Experimental Pathology, Wroclaw Medical University, Borowska 213, 50-556 Wroclaw, Poland; piotrdonizy@wp.pl; 5 Department of Ophthalmology, Institut Curie, 75005 Paris, France; nathalie.cassoux@curie.net; 6 Department of Ophthalmology, Wroclaw Medical University, 50-556 Wroclaw, Poland; mgajdzis@usk.wroc.pl; 7 Department of Biopathology, Institut Curie, PSL Research University, 75005 Paris, France; sophie.gardrat@curie.net

**Keywords:** HDAC, uveal melanoma, immunohistochemistry, prognosis

## Abstract

**Simple Summary:**

Histone Deacetylases (HDACs) have been reportedly associated with tumor development and progression in several types of human malignancy, being currently investigated as potential targets of anti-cancer therapy. The aim of this study is to assess the clinical significance and prognostic role of the of HDAC-1, -2, -4, and -6 immunohistochemical expression, in 75 uveal melanoma (UM) cases. HDACs are differentially expressed in UMs, HDAC-2 being the most frequently expressed isoform, whereas cytoplasmic expression of class I HDAC isoforms is also observed. Additionally, HDAC-1 was associated with increased tumor size, HDAC-6 with mitotic index, and HDAC-2 with epithelioid cell morphology and presence of tumor-infiltrating lymphocytes, both parameters of adverse prognosis. Moreover, our data support a significant association of HDAC-2 with patients’ improved OS. These findings suggest that HDACs, and especially HDAC-2, may be implicated in the formation and progression of UM.

**Abstract:**

Background: Uveal melanoma (UM) represents the most common primary intraocular malignancy in adults, exerting high metastatic potential and poor prognosis. Histone deacetylases (HDACs) play a key role in carcinogenesis, and HDAC inhibitors (HDACIs) are currently being explored as anti-cancer agents in clinical settings. The aim of this study was to evaluate the clinical significance of HDAC-1, -2, -4, and -6 expression in UM. Methods: HDAC-1, -2, -4, and -6 expression was examined immunohistochemically in 75 UM tissue specimens and was correlated with tumors’ clinicopathological characteristics, the presence of tumor-infiltrating lymphocytes (TILS), as well as with our patients’ overall survival (OS). Results: HDAC-2 was the most frequently expressed isoform (66%), whereas we confirmed in addition to the expected nuclear expression the presence of cytoplasmic expression of class I HDAC isoforms, namely HDAC-1 (33%) and HDAC-2 (9.5%). HDAC-4 and -6 expression was cytoplasmic. HDAC-1 nuclear expression was associated with increased tumor size (*p* = 0.03), HDAC-6 with higher mitotic index (*p* = 0.03), and nuclear HDAC-2 with epithelioid cell morphology (*p* = 0.03) and presence of tumor-infiltrating lymphocytes (*p* = 0.04). The association with the remaining parameters including Monosomy 3 was not significant. Moreover, the presence as well as the nuclear expression pattern of HDAC-2 were correlated with patients’ improved OS and remained significant in multivariate survival analysis. Conclusions: These findings provide evidence for a potential role of HDACs and especially HDAC-2 in the biological mechanisms governing UM evolution and progression.

## 1. Introduction

Epigenetic and post-translational modifications have been suggested to comprise an important regulatory mechanism of gene transcription, being increasingly correlated with carcinogenesis as well as with neoplastic disease progression. Core histones are known to undergo extensive post-translational modifications of their long N-terminal extensions such as acetylation, methylation, phosphorylation, ubiquitination, sumoylation, and ADP-ribosylation [1]. The acetylation of core histones is tightly regulated by the balance of two counteracting enzymatic activities: those of histone acetyl transferases (HATs) and of histone deacetylaces (HDACs) [2,3]. The latter remove acetyl groups from histone specific lysine residues, thereby increasing the charge density on the N-termini of the core histones, strengthening histone tail-DNA interactions, and blocking access of the transcriptional machinery to the DNA template, leading thus to transcriptional repression [3]. Deacetylation of a given lysine residue may also allow its further modification by histone methyltransferases [4].

The human HDAC protein family encompasses 18 different members divided into four classes according to phylogenetic analyses and sequence homologies with the yeast proteins Rpd3, Hos1 and Hos2 (class I), HDA1 and Hos3 (class II), and the sirtuins (class III) [5,6]. Class I and class II (IIa and IIb) proteins are evolutionarily related and share a common enzymatic mechanism, the Zn-catalyzed hydrolysis of the acetyl-lysine amide bond. Higher eukaryotes also express an additional Zn-dependent HDAC (HDAC-11) that is phylogenetically different from class I and class II enzymes and is regarded as a separate class (class IV). Class III proteins are unrelated to class I, II, or IV and catalyze the transfer of the acetyl group onto the sugar moiety of Nicotinamide adenine dinucleotide, namely NAD [6]. Among class I deacetylases (HDAC-1, -2, -3, and -8), HDAC-1 and -2 are the most closely related (82% sequence identity), seem to be involved in the regulation of cell cycle genes such as *p21*, and have been also reported to deacetylate non-histone substrates [1]. The class IIa HDACs, (HDAC-4, -5, -7, and -9), are associated with transcription factors, notably of the MEF and Runx families, and seem to control differentiation and cellular hypertrophy in muscle and cartilage tissues [1]. Class IIb HDACs (HDAC-6 and -10), have a duplication of their catalytic domains, whereas HDAC-6 is the only deacetylase known to act on tubulin, which is required for disposal of misfolded proteins in aggresomes [1]. HDAC proteins, and especially the most thoroughly investigated members of class I, have been recently shown to be overexpressed in a plethora of human malignancies, being associated also with tumorigenesis, disease progression, and prognosis [7].

Uveal melanoma (UM) represents the most common primary intraocular malignancy in adults, comprising approximately 3% of all melanoma cases. It arises from melanocytes along the uveal tract, including the iris, ciliary body, and choroid [8]. Approximately half of the patients present with metastatic disease and for these patients’ prognosis remains dismal, with a reported death rate of 80% at 1 year and 92% at 2 years [9,10]. Given that UM is an aggressive type of malignancy exhibiting a strong tendency for lethal metastasis, the understanding of its biological background and the development of targeted therapeutic agents and treatment approaches is necessary [11]. In the past decade, a family of enzymes that block the action of HDACs, namely histone deacetylase inhibitors (HDACIs), has generated increased interest due to their possible therapeutic role in uveal melanoma [12,13,14].

Apart from the increasing research of HDACIs in UMs, there is, to the best of our knowledge, to date, no comprehensive available information regarding the clinical significance of HDACs expression. Souri et al. recently described the expression of HDACs using gene expression profiling [15,16], while only one recent observational immunohistochemical study exists investigating the expression of HDACs in 16 UM cases [17]. In view of the above considerations, the present study aims to assess the immunohistochemical expression of several members of HDAC protein family classes I and II (a,b), namely HDAC-1, -2, -4, and -6, in UM specimens, in association with clinicopathological parameters as well as patients’ overall survival (OS) and disease-free survival (DFS).

## 2. Materials and Methods

### 2.1. Patients

This is a study of archival histopathological material from 75 patients with UM diagnosed in 2007–2008 at the Curie Institute, Paris, France, for whom medical records were available. All patients underwent a surgical enucleation and none of them had received any kind of prior radiotherapy or chemotherapy before surgery. The study was conducted in accordance with the Declaration of Helsinki, and it was approved by the Bioethics Committee of the National and Kapodistrian University of Athens, Greece (019/8.10.18). Due to the retrospective nature of the studies and the lack of impact on the treatment of patients, it was not necessary to obtain informed consent.

In this study, clinical parameters that are well known prognostic factors in UM were taken into account (age, tumor size, intra- or extra-scleral extension, grading, mitotic activity, chromosome 3 loss, and presence of metastasis) as well as three additional parameters that most strongly affect the visual acuity-tumor location in the posterior pole, retinal detachment, and vitreous hemorrhage. In a small percentage of cases (25/30) information regarding the presence of 8q gain was available. Tumor size was defined as the largest basal diameter (in mm). Cell type was evaluated by hematoxylin and eosin (H&E) staining according to the modified Callender classification system. Mitotic activity was assessed on ×400 in 40 fields using hematoxylin and eosin staining. The presence of tumor-infiltrating (TILS) and peritumoral lymphocytes (PLS) was evaluated in each case as appropriate. The clinicopathological characteristics of the cases included in this study are presented in Table 1.

### 2.2. Immunohistochemistry

Immunohistochemistry (IHC) for HDAC-1, -2, -4, and -6 was performed on formalin-fixed, paraffin-embedded tissue sections using the following antibodies: rabbit polyclonal anti-HDAC-1 (H-51, sc-7872, Santa Cruz Biotechnology, Santa Cruz, CA, USA), rabbit polyclonal anti-HDAC-2 (H-54, sc-7899, Santa Cruz Biotechnology, Santa Cruz, CA, USA), mouse monoclonal anti-HDAC-4 (A-4, sc-46672, Santa Cruz Biotechnology, Santa Cruz, CA, USA), and mouse monoclonal anti-HDAC-6 (D-11, sc-28386, Santa Cruz Biotechnology, Santa Cruz, CA, USA). Due to limited tumor tissue in some cases, there was available staining for HDAC-1 in 69 cases, HDAC-2 in 74 cases, HDAC-4 in 69 cases, and HDAC-6 in 72 cases. Immunostaining was performed using Autostainer 48 (DAKO, Glostrup, Denmark). Liquid Permanent Red (DAKO) was used as a detection system. Red chromogen visualization kit enabled visualization in tissues containing a large amount of melanin. Appropriate negative and positive controls were used as appropriate [18].

IHC evaluation was performed by counting at least 1000 tumor cells in each case independently by two experienced pathologists (S.T., J.K.), blinded to clinical information, with complete interobserver compliance. Nuclear and cytoplasmic immunoreactivity was evaluated separately. The extent of nuclear HDAC-1, -2, -4, and -6 expression was calculated by the percentage of positive tumor cells to the total number of tumor cells within each specimen and further categorized in four groups: 0 (no positive cells), 1 (<10% of positive cells), 2 (11–50% positive cells), 3 (51–80% positive cells) and 4 (>80% positive cells). The staining intensity was estimated in four categories: 0 (no reaction), 1 (mild reaction), 2 (moderate reaction), and 3 (intense reaction). An immunoreactive score (IRS) combining percentage of staining multiplied by the staining intensity was created (score 1–12) and then further categorized into four categories: negative expression (IRS 0–1), mild expression (IRS 2–3), moderated expression (IRS 4–8), and strong expression (IRS 9–10). Moreover, since HDAC-2 IHC expression exhibited an heterogenous staining pattern, we classified HDAC-2 immunoreactivity into isolated clusters of tumor cells, multiple clusters, and widespread expression throughout the tumor.

### 2.3. Statistical Analysis

Statistical analysis was performed by a MSc biostatistician (GL). The association between the IHC expression of HDAC-1, -2, -4, and -6 and clinicopathological characteristics was examined using non-parametric tests with correction for multiple comparisons, as appropriate. Survival analysis was performed using Kaplan–Meier survival curves and the differences between the curves were compared with log-rank test. Cox regression analysis was developed to evaluate to potential prognostic value of each parameter independent of the remaining available parameters. A *p*-value of <0.05 was considered statistically significant. A *p*-value of > 0.05 but lower of < 0.10 was considered of marginal significance. The analysis was performed with the statistical package STATA 11.0/SE (College Station, TX, USA) for Windows.

## 3. Results

### 3.1. Study Population

Thirty-one of the patients were men (41.3%) and 44 women (58.7%), with a median age at diagnosis 65 years (range 14–94 years). Tumor size varied significantly from 7 to 27 mm (median 15 mm). Cell type was categorized according to the modified Callender classification system as follows: 18 (24%) cases comprised of epithelioid cell melanomas, 21 (28%) of spindle cell melanomas, and the remaining 36 (48%) melanomas were of mixed-cell type. These were all cases of ciliary body and choroid UM, in six of which (8%) a per continuitatem involvement of the iris was also observed. The T-category according to AJCC was as follows: T1, 1 case (1%), T2, 11 cases (25%), T3, 25 cases (33%); and T4, 38 cases (51%). Forty patients (53%) had a metastatic disease at the time of diagnosis. Thirteen patients (13/57, 23%) had a loss of chromosome 3 and 25 (25/30, 83%) a gain of chromosome 8. Forty-three (57%) patients died from their disease within 9–99 months (median value 29 months). The remaining 32 patients were followed up for a median period of 65 (range 5–115 months). seven patients had a relapse of their disease in a median follow up of 53 months (13–109 months).

### 3.2. HDAC-1 IHC Expression and Association with Clinicopathological Parameters

**Figure 1:**
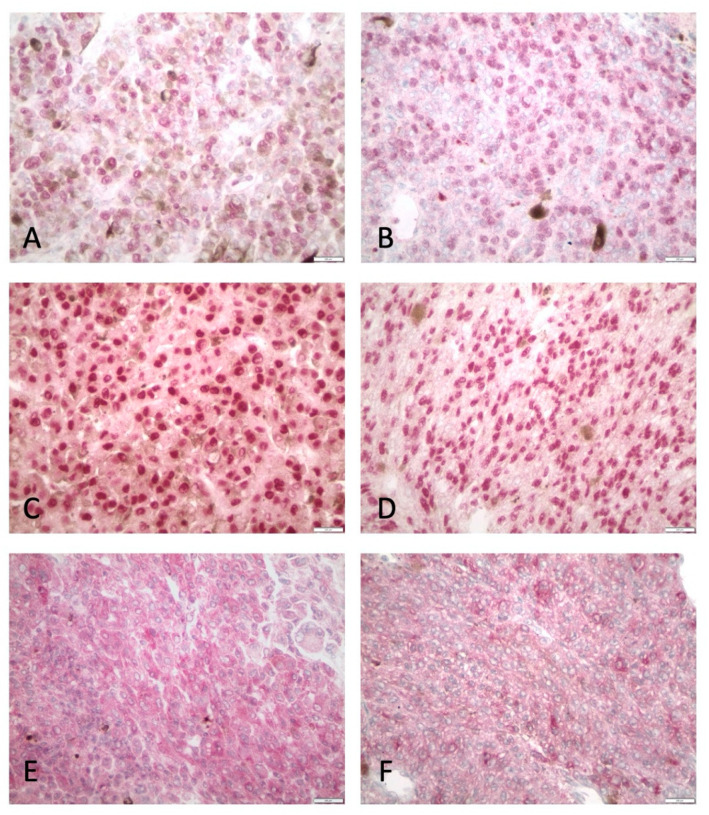
Immunohistochemical expression of HDAC-1, -2, -4 and -6 in UMs. (**A**) Nuclear HDAC-1 expression, (**B**) nuclear and cytoplasmic HDAC-2 expression, (**C**) strong HDAC-2 expression in epithelioid-cell UM, (**D**) HDAC-2 expression in spindle-cell UM, (**E**) cytoplasmic HDAC-4 expression, (**F**) cytoplasmic HDAC-6 expression (×200).

HDAC-1 exhibited both nuclear and cytoplasmic expression (Figure 1, Table 2). Nuclear HDAC-1 expression was observed in 18 (26%) and cytoplasmic in 23 (33%) of the examined cases. The majority of positive cases displayed a mild cytoplasmic (18/23, 78%) and a moderate nuclear (11/18, 61%) HDAC-1 IRS.

**Figure 2:**
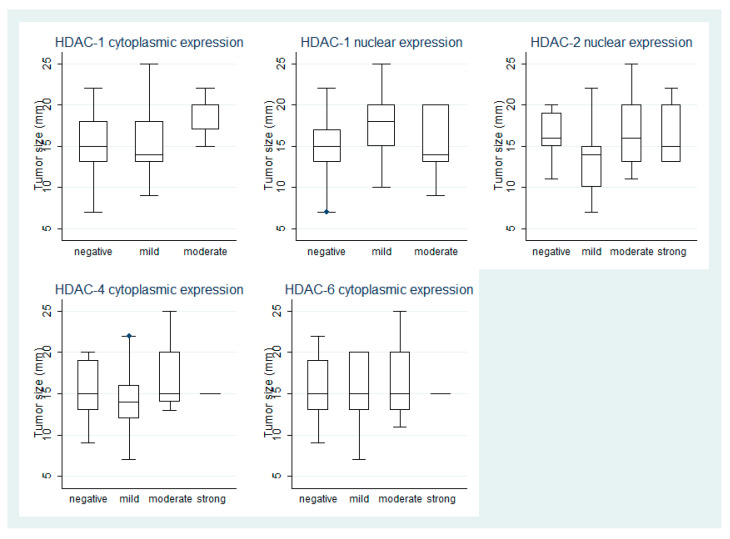
Schematic representation of the associations between HDAC-1, -2, -4, and -6 expression with tumor size.

Moderate cytoplasmic HDAC-1 IRS was observed in cases with increased tumor size (Mann–Whitney U test, *p* = 0.03, Figure 2) and marginally more frequently in cases with gain of chromosome 8q (40% vs. 4% Fischer’s exact test, *p* = 0.07).

The correlations between HDAC-1 IRS nuclear or cytoplasmic and TILS, PLS and the remaining clinicopathological parameters, i.e., monosomy 3, were not significant (*p* > 0.10). The associations of HDAC-1 nuclear and cytoplasmic IRS with clinicopathological parameters are presented in Supplementary Table S1.

### 3.3. HDAC-2 IHC Expression and Association with Clinicopathological Parameters

HDAC-2 expression was mainly nuclear, with only a few cases (7/74, 9.5%) displaying also cytoplasmic immunoreactivity (Figure 1, Table 2). Nuclear HDAC-2 expression was observed in 49 (66%) of the 74 examined cases. A strong nuclear HDAC-2 IRS was observed in seven (9.5%) cases, whereas the remaining 42 cases showed either a mild (26%) or a moderate (31%) IRS. Moreover, the vast majority of the cases with moderate/strong HDAC-2 nuclear IRS showed multiple clusters and widespread expression throughout the tumor (90%, 27/30), whereas only 16/44 (36%) of cases with absent/mild HDAC-2 IRS exhibited this pattern of staining (Fisher’s exact test, *p* < 0.0001). Due to the small number of cases displaying cytoplasmic expression, the analysis for the associations with clinicopathological features was performed only for nuclear HDAC-2 IRS.

Patients with negative or mild HDAC-2 nuclear IRS were older than patients with moderate or strong expression (Mann–Whitney U test, median values 68 years vs. 59.5 years, respectively, *p* = 0.05). A significant difference in the size of tumor was observed between the four levels of HDAC-2 nuclear expression according to IRS (Kwallis ANOVA *p* = 0.01, Figure 2). The same applies to different histological types, a moderate or strong expression being observed in 61% (11/18) of cases with epithelioid cell, in 42% (15/36) of cases with mixed-cell, and in only 20% (4/20) of cases with spindle cell morphology (Fischer’s exact test, *p* = 0.03). Similarly, a moderate or strong HDAC-2 cytoplasmic IRS was observed in 22% (4/18) of cases with epithelioid cell, in 3% (1/36) of cases with mixed-cell and in 10% (2/20) of cases with spindle cell morphology (Fischer’s exact test, *p* = 0.04). Moreover, the presence of nuclear HDAC-2 IRS was found in 72% (18/25) of cases with 8q gain, the respective value for cases without gain of chromosome 8q being only 20% (1/5) (Fischer’s exact test, *p* = 0.05).

HDAC-2 nuclear IRS was frequently higher in cases with brisk TILS when compared with cases with absent/non brisk TILS (*p* = 0.04 Fischer’s exact test). Moreover, HDAC-2 nuclear was frequently higher in cases with moderate amounts of PLS (72.7%), followed by cases with no PLS (37%), and finally, cases with high amounts of PLS (17%) (*p* = 0.04 Fischer’s exact test). The pattern of HDAC-2 staining was not correlated with any of the clinicopathological parameters.

The correlations between HDAC-2 IRS and the remaining clinicopathological parameters, i.e., monosomy 3 were not significant (*p* > 0.10). The associations of nuclear HDAC-2 IRS with clinicopathological parameters are presented in Supplementary Table S1.

### 3.4. HDAC-4 IHC Expression and Association with Clinicopathological Parameters

HDAC-4 expression was cytoplasmic in 31 of the examined 69 cases (45%) (Figure 1, Table 2). The majority of positive cases displayed a moderate or strong HDAC-4 expression (18/31, 58%).

**Figure 3:**
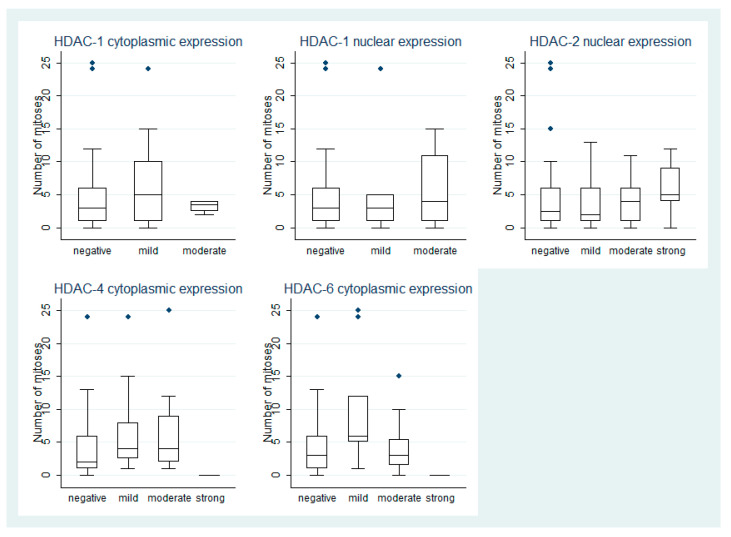
Schematic representation of the associations between HDAC-1, -2, -4, and -6 expression with number of mitoses.

HDAC-4 IRS displayed a borderline correlation with mitotic index, cases with positive staining showing marginally increased number of mitoses when compared to negative cases (Kruskal–Wallis ANOVA *p* = 0.05, Figure 3). The correlations between HDAC-4 IRS and TILS, PLS and the remaining clinicopathological parameters, i.e., monosomy 3 were not significant (*p* > 0.10). The associations of cytoplasmic HDAC-4 IRS with clinicopathological parameters are presented in Supplementary Table S1.

### 3.5. HDAC-6 IHC Expression and Association with Clinicopathological Parameters

HDAC-6 exhibited cytoplasmic expression in 35 of the 72 examined cases (49%, Figure 1, Table 2). The majority of positive cases displayed a moderate staining (25/35, 7%).

A significant difference in the number of mitoses was found between the four levels of HDAC-6 expression according to IRS (Kruskal–Wallis ANOVA *p* = 0.03, Figure 3). Moreover, all the cases that displayed an involvement of iris per continuitatem showed HDAC-6 immunoreactivity (6/72, 8%) (Chi square test, *p* < 0.01).

The correlations between HDAC-6 IRS and TILS, PLS, and the remaining clinicopathological parameters, i.e., monosomy 3, were not significant (*p* > 0.10). The associations of cytoplasmic HDAC-6 IRS with clinicopathological parameters are presented in Supplementary Table S1.

### 3.6. Survival Analysis

**Figure 4:**
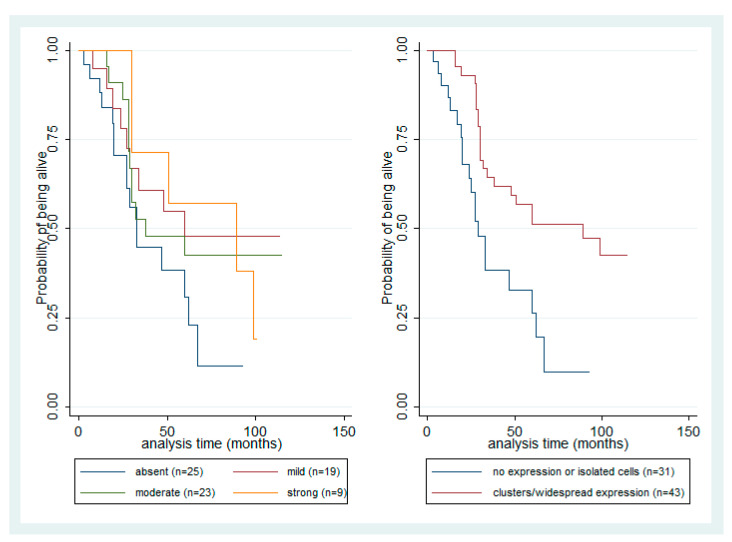
Kaplan–Meier survival curves according to four levels of HDAC-2 nuclear IRS (absent, mild, moderate, strong) and the patter of heterogeneous expression of HDAC-2 in UMs.

Survival analysis was performed in order to assess the association of HDAC-1, -2, -4, and -6 expression with OS and DFS. Increased tumor size (*p* = 0.01), presence of metastasis (*p* < 0.001), presence of epithelioid cell morphology (*p* = 0.008), involvement of iris (*p* = 0.02), ciliary body involvement (*p* = 0.02), absence of intrascleral location (*p* = 0.02) and presence of extrascleral location (*p* = 0.09) adversely affected OS, the latter association of borderline significance. HDAC-2 nuclear expression examined in the four levels of IRS did not seem to have a significant association with survival (log rank test *p* > 0.10, Figure 4). However, the presence of positive nuclear HDAC-2 expression and the pattern of HDAC-2 nuclear staining showing isolated clusters of tumor cells were observed to be associated with favorable patients’ prognosis (log rank test, *p* = 0.04 and *p* = 0.001, respectively, Figure 4). Accordingly, the presence of cytoplasmic HDAC-2 expression was marginally associated with a better OS (log rank test, *p* = 0.08). None of the investigated parameters showed any correlation with DFS.

In a multivariate survival analysis, we adjusted different Cox proportional hazards models for HDAC-2 nuclear IRS and heterogeneous pattern of HDAC-2 nuclear to avoid collinearity since the two parameters were strongly associated. In each model, we included all parameters that were proven to be significant in univariate survival analysis for which we had information in the whole number of patients. Both nuclear HDAC-2 IRS (HR = 0.301, *p* = 0.001, Table 3, Model A) and pattern of staining (HR = 0.286, *p* = 0.001, Table 3, Model B) remained significant, and thus were proven to be independent factor of favorable prognosis. We also adjusted separate models for HDAC-1, -4, and -6 IRS; however, these failed to show any significant correlation with survival (Supplementary Table S2, Models A–D).

## 4. Discussion

In the last decade, HDACs have been suggested to play a crucial role in the regulation of cell proliferation, differentiation, and apoptosis in various human hematological, solid, and mesenchymal malignancies [18,19,20,21,22,23,24,25,26]. HDAC IHC expression has been described in several tumor types, being associated with important clinicopathological parameters, including patients’ prognosis [18,19,20,22,24,25,26]. Information on HDAC IHC expression in UMs remains scarce, as the available data so far are limited to one recent study conducted on 16 UM specimens without, however, any attempt to correlate it with patients’ clinical information [17]. Moreover, Souri et al., in two recent studies, report the expression of HDAC-1, -3, -4, -8 using gene expression profiling [15,16]. The present study assessed the clinical significance of HDAC-1, -2, -4, and -6 immunohistochemical expression in UMs.

In this study, we observed a nuclear HDAC-1 and -2 staining, in consistency with previous reports and in keeping with the fact that class I HDACs are reported to be ubiquitously located in cell nucleus, due to a lack of a nuclear export signal [27]. We also observed a cytoplasmic HDAC-1 and -2 immunoexpression in 34.8% and 9.5% of the examined cases, respectively. Even though the respective literature mostly focuses on the role of class I HDACs in the nucleus, cytoplasmic immunoreactivity has also been reported, the function of which remains unclear. In this context, it has been suggested that the NFkappaB inhibitor, IkappaBα, interacts with HDAC-1 and HDAC-3 in Hela cells, sequestering these proteins in the cytoplasm [28]. HDAC-1 and -2 cytoplasmic staining has been reported in various normal tissues, such as in pancreatic and hepatic parenchyma, small bowel, and gastric mucosa, and in non-tumoral ocular structures [17,21], as well as in malignant neoplasms, such as salivary gland tumors, alveolar soft part sarcoma, and neuroblastoma [18,21,29]. On the other hand, HDAC-4 and -6 exhibited cytoplasmic immunoreactivity, in keeping with the reported staining patterns of these proteins. Class II HDAC proteins are, according to the literature, capable of nucleocytoplasmic shuttling in response to certain cellular signals [26].

In accordance with the findings of Levinzon et al. [17], HDAC-2 had the strongest expression among the examined HDACs in our cohort, being expressed in 66% of the examined cases and exhibiting a moderate to strong expression in 61% of the positive cases. Interestingly, HDAC-2 staining demonstrated an heterogenous pattern, varying between isolated clusters of tumor cells to multiple clusters and to widespread expression throughout the tumor, which was strongly correlated with IRS. HDAC-4 and -6 were expressed in approximately half the examined cases (45% for HDAC-4 and 49% for HDAC-6), whereas HDAC-1 was the least expressed HDAC- protein in our cohort, being positive in about one-third of the examined cases. Similar findings regarding HDAC-1 have been reported in skin melanoma and in mesenchymal tumors, suggesting that HDAC-2 is the class I isoform more likely associated with the pathogenesis of UMs [21,30]. Moreover, we found moderate/strong HDAC-4 and -6 expression in a significant part of the positive cases (18/31, 58% for HDAC-4 and 26/35, 74% for HDAC-6), in line with previous findings of Levinzon et al. regarding HDAC-4, who suggested that this finding can be associated with HDAC-4 upregulation in malignancies with excess inflammation and chemokine signaling, such as UM [17].

Regarding the correlations of HDACs with clinicopathological parameters, elevated HDAC expression in general has been mainly associated with increased tumor size and aggressive phenotype [18]. Tumor size has been one of the most important clinical prognostic features of UMs [30]. In our cohort, increased cytoplasmic HDAC-1 expression was indeed correlated with increased tumor size, whilst the correlation of HDAC-2 immunoreactivity with tumor size was not straightforward. In particular, cases with mild HDAC-2 nuclear expression seemed to have the smaller tumor size in comparison to the rest expression levels. Moreover, we observed that HDAC-2 expression was correlated with younger patients’ age. A similar correlation has also been reported in mobile tongue squamous cell carcinoma [20], although there are reports of a positive correlation between patients’ age and HDACs expression in other types of malignancies, such as gastric adenocarcinoma [26]. According to the literature, however, age at the time of diagnosis does not seem to affect patients’ prognosis in UMs [30,31,32].

Importantly, HDAC-2 expression was associated with tumor histological type, being higher in the epithelioid cell than in spindle cell morphology. A similar association regarding the expression of HDAC-1 by gene expression profiling has been reported by Souri et al. [16]. Tumor cell type has been considered an important prognostic factor for patients with UMs. According to the modified Callender classification, it has been traditionally considered to have a strong correlation with mortality; spindle cell UMs seem to have the best prognosis, mixed cell type UMs an intermediate prognosis, and epitheloid cell type UMs the worst prognosis [30,32,33], a finding that was also recapitulated in our cohort in our survival analysis. In contrast to the previously reported results by Souri et al. [16], in our cohort, only HDAC-2 immunoexpression was correlated with the presence of ILS, being higher in cases with a small number of TILS, which is also considered to be another factor of poor prognosis [30].

Moreover, increased HDAC-2 and marginally cytoplasmic HDAC-1 expression were correlated with the presence of Chromosome 8q gain. Alterations of Chromosome 8 are common in UMs, 8q gain being the most common (occurring in 41 to 53% cases) (in form of trisomy 8, isochromosome 8q, and amplification of the c-myc gene), while 8p loss rarely occurs [32]. Chromosome 8q gain is considered an important adverse prognostic factor for UM, either when it presents alone or co-exists with monosomy 3 [34]. It has been recently suggested that HDAC-1, -3, -4, and -8 expression is higher in UMs with monosomy 3 [15], a relationship which however was not reproduced in our cohort, perhaps due to the decreased number of cases for which information about the presence of monosomy 3 was available.

Both class II HDACs isoforms that were investigated in our study showed a correlation with the tumor mitotic index, the latter being significantly associated with increased mortality rate [30]. In particular, the higher mitotic indexes were observed in cases with mild HDAC-6 expression, in contrast to cases without HDAC-6 expression. In addition, HDAC-4 expression was higher in cases with increased mitotic activity, but this relationship was of marginal significance. Relevant to these findings are previous observations in mouse models with subcutaneous xenografts from implanted A375 cell lines, in which HDAC-6 knock down suppressed proliferation and induced apoptosis [30].

An important finding emerging from the present study is that the presence of HDAC-2 expression in UMs connotes a better survival probability. To the best of our knowledge, this appears to be the first report investigating the potential prognostic role of HDACs in UMs. A similar borderline association between cytoplasmic HDAC-2 expression and OS was observed. HDAC-1 expression does not seem to be informative in this regard. According to a comprehensive review of Weichert [24], class I HDAC isoforms are mostly expected to be associated with poor patients’ survival. However, there are recently increasing reports of a correlation with favorable prognosis in some tumor types, such as pancreatic, colorectal, salivary gland, and invasive breast carcinomas not otherwise specified (NOS) [18,19,22,23]. Interestingly, although HDAC-2 expression was associated with clinicopathological parameters suggesting poor patients’ prognosis, it emerged in survival analysis as a potential favorable prognosticator. The fact that the presence of nuclear HDAC-2 expression remains in combination with lower tumor size, absence of metastatic disease, and spindle cell morphology, an independent factor of better prognosis in multivariate survival analysis, implicates a possible unknown intrinsic role of HDAC-2 in a patients’ prognosis, which cannot be attributed to its association with these parameters. In contrast to HDAC class I isoforms, class II isoforms are mostly associated with better OS [24]. However, in our study, they did not seem to convey any important prognostic information. The results of survival analysis recapitulate many of the traditional parameters that have been proposed as important determinants of clinical outcome in UMs, namely tumor size, presence of metastasis, and histological type [30,32,33], supporting the validity of statistical analysis and denoting that our cohort, although relatively small, is representative.

In the past few years, numerous clinical trials investigating the role of HDACIs alone or in combination with other drugs in numerous malignancies have been conducted, showing encouraging anti-tumor effects [35]. Four HDACIs have already been approved for the treatment of cutaneous and peripheral T-cell lymphoma as well as multiple myeloma [36]. Several HDACIs have been studied in UM cell lines with promising results [15]. In particular, the inhibition of proliferation, cell-cycle arrest, induction of apoptosis, inhibition of migration, induction of morphologic differentiation, and transition from a high-risk to a low-risk gene-expression profile are some of the antitumor effects of HDACIs on UM cells [13]. Currently, there are HDACIs that are tested as monotherapy against UM (vorinostat, NCT01587352), whereas a combination of pembrolizumab with class I HDACI entinostat is currently under investigation in a phase 2 clinical trial (NCT01814046) [37]. Although HDACIs are recognized as one of the most promising targets, additional studies remain to determine the efficiency of these therapies.

## 5. Conclusions

The present study shows that HDACs are differentially expressed in UMs, HDAC-2 being the most frequently expressed isoform, whereas cytoplasmic expression of class I HDAC isoforms is also observed. Additionally, this is the first study to report associations of elevated HDACs IHC expression with several clinicopathological parameters. HDAC-1 was associated with increased tumor size, HDAC-6 with mitotic index, and HDAC-2 with epithelioid cell morphology, presence of ILS, and gain of chromosome 8q, all parameters of adverse prognosis. Of even more clinical significance are the data supporting the association of HDAC-2 with patients’ improved OS. These findings provide evidence for a potential role of HDACs in the biological mechanisms governing UM evolution and progression. Further studies offering a deeper understanding on the potential role of these molecules and consequently of the mechanisms involved in the antitumor activity of HDACIs are warranted.

## Figures and Tables

**Table 1:** Clinicopathological characteristics of 75 patients with UM (* per continuitatem, ** presence of disease after a disease-free period of time following initial treatment).

Parameter	Median	Range
**Age**	65	14–94 years
**Number of Mitoses per 40 HPFs**	3	0–25
**Tumor size**	15	range 7–25 mm
	**Number**	%
**Gender**		
Male	31/75	41%
Female	44/75	59%
**Posterior pole involvement**	18/75	26%
**Ciliary body involvement**	32/75	43%
Iris involvement *	6/75	8%
**Irido-corneal angle involvement**	7/75	9%
**Presence of retinal detachment**	32/75	43%
**Presence of vitreous hemorrhage**	10/75	13%
**Intrasclera involvement**	64/75	85%
**Extrasclera involvement**	8/75	12%
**Histological cell type**		
Epithelioid cell	18/75	24%
Mixed cell	36/75	48%
Spindle cell	21/75	28%
**Loss of chromosome 3**	13/57	23%
**Gain 8q**	25/30	83%
**Presence of metastasis**	40/75	53%
**T-category (AJCC)**		
Τ1	1/75	1%
Τ2	11/75	15%
Τ3	25/75	33%
Τ4	38/75	51%
**Event**		
Death of disease	43/75, within 9–99 months	57%
Censored	32/75, follow-up 5–115 months	43%
Presence of relapse **	7/75, within 13–109 months	9%

**Table 2:** Immunohistochemical expression of HDAC-1, -2, -4, and -6 in UMs.

Parameter	Immunoreactivity Score (IRS)			
	Absent	Mild	Moderate	Strong
**HDAC-1**				
Nuclear	51 (74%)	7 (10%)	11 (16%)	-
Cytoplasmic	46 (67%)	18 (26%)	5 (7%)	-
**HDAC-2**				
Nuclear	25 (34%)	19 (26%)	23 (31%)	9 (9%)
Cytoplasmic	67 (91%)	3 (4%)	4 (5%)	-
**HDAC-4 cytoplasmic**	38 (55%)	13 (19%)	17 (25%)	1 (1%)
**HDAC-6 cytoplasmic**	37 (51%)	9 (12%)	25 (36%)	1 (1%)

**Table 3:** Cox proportional hazards models in UMs including HDAC-nuclear IRS and pattern of heterogeneous staining.

**Model A**	**Hazard Ratio (HR)**	***p***	**95% Confidence Interval**	
HDAC-2 nuclear IRS	0.286	0.001	0.133	0.618
Tumor size	1.133	0.02	1.023	1.255
Number of mitoses	1.053	0.07	0.997	1.113
Mixed cell type vs. Epithelioid type	0.605	0.22	0.271	1.351
Spindle cell type vs. Epithelioid type	0.169	0.003	0.052	0.545
Presence of metastasis	3.949	0.002	1.682	9.271
**Model B**	**Hazard Ratio (HR)**	***p***	**95% Confidence Interval**	
HDAC-2 heterogeneous staining widespread/clusters vs. negative/isolated cells	0.303	0.001	0.145	0.631
Tumor size	1.078	0.16	0.972	1.194
Number of mitoses	1.051	0.07	0.995	1.110
Mixed cell type vs. Epithelioid type	0.567	0.17	0.250	1.284
Spindle cell type vs. Epithelioid type	0.215	0.007	0.070	0.662
Presence of metastasis	3.610	0.003	1.555	8.381

## Data Availability

The data presented in this study are available on request from the corresponding author.

## Supplementary Materials

The following are available online at https://www.mdpi.com/article/10.3390/cancers13194763/s1, Table S1; Associations between HDAC-1, -2, -4 and -6 IRS with clinicopathological features. Results of Fischer’s exact test. Table S2: Cox proportional Hazards models in UMs for HDAC-1, -4 and -6.
