# Supplementary material for: Histone Deacetylase (HDAC)-1, -2, -4, and -6 in Uveal Melanomas: Associations with Clinicopathological Parameters and Patients’ Survival
Source: Cancers (Basel). 2021 Sep 23;13(19):4763. doi: 10.3390/cancers13194763 (PMC8507547; doi:10.3390/cancers13194763)
Supplement: Supplementary file 1 [file cancers-13-04763-s001.zip › cancers-1377023-supplementary.pdf]

Supplementary Table S1. Table Associations between HDAC−1, −2, −4 and −6 IRS with clinicopathological features. Results of Fischer's exact test.

	HDAC−1 nuclear expression			HDAC−1 cytoplasmic expression			HDAC−2 nuclear expression			HDAC−4 cytoplasmic expression			HDAC−6 cytoplasmic expression		
	Absent/ mild	Mod./ strong	p	Absent/ mild	Mod./ strong	p	Absent/ mild	Mod./ strong	p	Absent/ mild	Mod./ strong	p	Absent/ mild	Mod./ strong	p
	# of cases			# of cases			# of cases			# of cases			# of cases		
**Gender**															
Male	24	5	>0.99	25	4	0.15	16	14	0.47	20	9	0.58	18	12	0.623
Female	34	6		39	1		28	16		31	9		28	14	
**Presence of retinal detachment**															
No	35	6	0.75	37	4	0.64	25	17	>0.99	29	11	0.79	29	13	0.33
Yes	23	5		27	1		19	13		22	7		17	13	
**Presence of vitreous hemorrhage**															
No	49	10	>0.99	54	5	>0.99	40	25	0.47	45	14	0.44	41	21	0.48
Yes	9	1		10	0		4	5		6	4		5	5	
**Histological cell type**															
Epithelioid cell	14	1	0.39	13	2	0.42	7	11	0.03	10	4	0.39	9	8	0.46
Mixed cell	28	5		32	1		21	15		24	11		24	10	
Spindle cell	16	5		19	2		16	4		17	3		13	8	
**Loss of chromosome 3**															
No	12	1	>0.99	12	1	>0.99	7	6	0.75	7	4	0.72	8	5	0.74
Yes	33	5		34	4		27	17		28	12		28	13	
**Gain 8q**															
No	5	0	>0.99	3	2	0.07	4	1	>0.99	4	1	0.63	3	2	0.63
Yes	22	2		23	1		17	8		15	9		17	7	
**Presence of metastasis**															
No	27	7	0.34	32	2	>0.99	22	12	0.48	24	10	0.59	20	14	0.46
Yes	31	4		32	3		22	18		27	8		26	12	
**TILS**															
absent/non brisk	54	10	>0.99	60	4	0.32	43	25	0.04	48	17	>0.99	44	22	0.18
brisk	4	1		4	1		1	5		3	1		2	4	
**PLS**															
Absent	44	10	0.85	51	3	0.17	36	21	0.04	41	14	0.88	39	18	0.23
Moderate	9	1		8	2		3	8		6	3		4	6	

High	5	0		5	0		5	1		4	1		3	2	
T-category															
T1	0	1	0.25	1	0	0.17	1	0	0.14	1	0	0.55	0	1	0.51
T2	10	1		11	0		9	2		9	1		8	3	
T3	18	4		22	0		16	9		18	6		14	10	
T4	30	5		30	5		18	19		23	11		24	12	

Supplementary Table S2. Cox proportional Hazards models in UMs for HDAC−1, −4 and −6.

**Model A**	**Hazard ratio (HR)**	**P**	**95% confidence interval**	
HDAC−1 cytoplasmic IRS	1.107	0.90	0.242	5.067
Tumor size	1.101	0.08	0.990	1.225
Number of mitoses	1.056	0.09	0.991	1.126
Mixed cell type vs Epithelioid type	1.027	0.95	0.447	2.360
Spindle cell type vs Epithelioid type	0.441	0.13	0.152	1.282
Presence of metastasis	2.899	0.01	1.242	6.765
**Model B**	**Hazard ratio (HR)**	**P**	**95% confidence interval**	**Hazard ratio (HR)**
HDAC−1 nuclear IRS	0.650	0.39	0.244	1.728
Tumor size	1.103	0.07	0.993	1.225
Number of mitoses	1.057	0.08	0.994	1.125
Mixed cell type vs Epithelioid type	1.106	0.81	0.485	2.525
Spindle cell type vs Epithelioid type	0.457	0.15	0.159	1.317
Presence of metastasis	2.862	0.01	1.240	6.604
**Model C**	**Hazard ratio (HR)**	**P**	**95% confidence interval**	
HDAC−4cytoplasmic IRS	0.818	0.60	0.382	1.751
Tumor size	1.134	0.02	1.021	1.260
Number of mitoses	1.046	0.18	0.980	1.116
Mixed cell type vs Epithelioid type	0.614	0.21	0.288	1.310
Spindle cell type vs Epithelioid type	0.227	0.01	0.076	0.679
Presence of metastasis	3.199	0.01	1.389	7.367
**Model D**	**Hazard ratio (HR)**	**P**	**95% confidence interval**	

HDAC−6 cytoplasmic IRS	1.074	0.85	0.503	2.295
Tumor size	1.097	0.08	0.989	1.217
Number of mitoses	1.052	0.12	0.988	1.121
Mixed cell type vs Epithelioid type	0.987	0.98	0.450	2.164
Spindle cell type vs Epithelioid type	0.407	0.09	0.145	1.142
Presence of metastasis	3.091	0.01	1.283	7.445
